# Inferior Colliculus Transcriptome After Status Epilepticus in the Genetically Audiogenic Seizure-Prone Hamster GASH/Sal

**DOI:** 10.3389/fnins.2020.00508

**Published:** 2020-05-26

**Authors:** Sandra M. Díaz-Rodríguez, Daniel López-López, Manuel J. Herrero-Turrión, Ricardo Gómez-Nieto, Angel Canal-Alonso, Dolores E. Lopéz

**Affiliations:** ^1^Institute of Neurosciences of Castilla y León, University of Salamanca, Salamanca, Spain; ^2^Institute of Biomedical Research of Salamanca, University of Salamanca, Salamanca, Spain; ^3^Department of Cellular Biology and Pathology, University of Salamanca, Salamanca, Spain; ^4^Neurological Tissue Bank INCYL (BTN-INCYL), Salamanca, Spain; ^5^BISITE Research Group, University of Salamanca, Salamanca, Spain

**Keywords:** audiogenic seizure, GASH/Sal, metabolomic, RNA-seq, RT-qPCR

## Abstract

The Genetic Audiogenic Seizure Hamster from Salamanca (GASH/Sal), an animal model of reflex epilepsy, exhibits generalized tonic–clonic seizures in response to loud sound with the epileptogenic focus localized in the inferior colliculus (IC). Ictal events in seizure-prone strains cause gene deregulation in the epileptogenic focus, which can provide insights into the epileptogenic mechanisms. Thus, the present study aimed to determine the expression profile of key genes in the IC of the GASH/Sal after the status epilepticus. For such purpose, we used RNA-Seq to perform a comparative study between the IC transcriptome of GASH/Sal and that of control hamsters both subjected to loud sound stimulation. After filtering for normalization and gene selection, a total of 36 genes were declared differentially expressed from the RNA-seq analysis in the IC. A set of differentially expressed genes were validated by RT-qPCR showing significant differentially expression between GASH/Sal hamsters and Syrian control hamsters. The confirmed differentially expressed genes were classified on ontological categories associated with epileptogenic events similar to those produced by generalized tonic seizures in humans. Subsequently, based on the result of metabolomics, we found the interleukin-4 and 13-signaling, and nucleoside transport as presumably altered routes in the GASH/Sal model. This research suggests that seizures in GASH/Sal hamsters are generated by multiple molecular substrates, which activate biological processes, molecular processes, cellular components and metabolic pathways associated with epileptogenic events similar to those produced by tonic seizures in humans. Therefore, our study supports the use of the GASH/Sal as a valuable animal model for epilepsy research, toward establishing correlations with human epilepsy and searching new biomarkers of epileptogenesis.

## Introduction

Epilepsy is one of the most common neurological disorders, affecting approximately one percent of the population. Although the etiology of epilepsy can be structural, genetic, infectious, metabolic, immune or unknown (last ILAE definition, Scheffer et al., 2017), genetic epilepsy accounts for one-third of all patients with epilepsy (Scheffer et al., 2017). In recent decades, an increasing number of epilepsy associated mutations have been identified, mainly in rare monogenic epileptic syndromes; however, only 1 to 2 % of idiopathic epilepsies appear to be monogenic (Lopes-Cendes and Oliveira Ribeiro, 2013). Examples of monogenic epileptic syndromes are autosomal dominant nocturnal frontal lobe epilepsy and progressive myoclonic epilepsy, in which, mutations of a single gene are sufficient to produce epileptic seizures (Lopes-Cendes and Oliveira Ribeiro, 2013).

Among the most commonly used and well characterized *in vivo* genetic models of epilepsy are the so-called genetically audiogenic seizure models, those with reflex epilepsy induced by high-intensity acoustic stimulation (Ross and Coleman, 2000; Kandratavicius et al., 2014; Garcia-Cairasco et al., 2017; Muñoz et al., 2017). This predisposition to seizures has enabled researchers to use audiogenic models of epilepsy in a wide range of studies on cellular and molecular activity, behavior, epilepsy comorbidities, development of new drugs, and ictogenic processes (Kandratavicius et al., 2014).

Among these models, the Genetic Audiogenic Seizure Hamster from Salamanca (GASH/Sal), developed and maintained at the Animal Experimentation Service of the University of Salamanca, exhibits an autosomal recessive pattern of heredity with audiogenic susceptibility (Muñoz et al., 2017). As occurs in other animal models of audiogenic epilepsy, the inferior colliculus (IC) is crucial for the initiation and propagation of audiogenic seizures in the GASH/Sal (Kesner, 1966; Wada et al., 1970; Faingold, 2004; Muñoz et al., 2017). These animals reach their maximum degree of seizure susceptibility between the second and fourth month of life, which then gradually disappears (Muñoz et al., 2017), and their seizures have been characterized as complete sound-evoked reflex seizures (Carballosa-Gonzalez et al., 2013). Furthermore, several studies have reported the inheritance pattern (Muñoz et al., 2017), and the neuroanatomical substrates underlying audiogenic seizure susceptibility (Sánchez-Benito et al., 2017, 2020) as well as the anticonvulsant effects after antiepileptic drug administration (Barrera-Bailón et al., 2013, 2017). It has also been found that the GASH/Sal exhibits altered gene expression of early growth response genes 1 to 3 (*Egr1, Egr2*, and *Egr3*) in the IC, presumably as an effect of stress associated to seizures (López-López et al., 2017).

The present study evaluates the global gene expression profiling of the IC in the GASH/Sal with sound-induced seizures when compared to control animals that received the same acoustic stimulation. To do so, we carried out a comparative transcriptome analysis of the IC in the GASH/Sal and matched control hamsters after loud sound stimulation. Our RNA-Seq findings showed that audiogenic seizures disrupted the gene expression in the IC of GASH/Sal hamsters, as validated by quantitative reverse transcription real-time PCR (RT-qPCR) for a specific set of genes. These results are of importance for searching common link to this heterogeneous disease in humans, with potential applications for diagnostic and therapeutic approaches in the clinical context.

## Methodology

### Animals

A total of 30, 3-month-old male Syrian hamsters (*Mesocricetus auratus*) were used in this study, namely 18 GASH/Sal hamsters from the inbred strain maintained at the animal’s facility of the University of Salamanca (Salamanca, Spain), and 12 controls, that is, RjHan:AURA Syrian hamsters, from Janvier Labs (Le Genest-Saint-Isle, France). The animals were subdivided into three groups: (1) The acoustically stimulated control group (Syrian control hamster stimulated, *n* = 12). All control hamsters exhibited absence of seizures after loud acoustic stimulation. (2) The acoustically stimulated GASH/Sal (GASH/Sal Stim; *n* = 12), corresponding to seizure-prone animals that were subjected to loud acoustic stimulation and presented generalized tonic–clonic seizures and clonic spasms. (3) The naïve GASH/Sal group (*n* = 6), corresponding to seizure-prone animals that did not receive any loud acoustic stimulation, and hence showed absence of audiogenic seizures.

The control and GASH/Sal animals that were exposed to loud sound stimulation were individually placed within an acrylic cylinder to receive a single high-intensity acoustic stimulus for 10 s. The stimulus used in the high-intensity acoustic stimulation protocol was recorded using a high-pass filter (N500 Hz; microphone Bruel and Kjaer #4134 and preamplifier Bruel and Kjaer #2619), digitized above 4 kHz, and reproduced by a computer coupled to an amplifier (Fonestar MA-25T, Revilla de Camargo, Spain) and a tweeter (Beyma T2010, Valencia, Spain) in the upper portion of the arena. The delivered sound was a semirandom acoustic stimulus of 0–18 kHz with an intensity of 115 to 120 dB (Barrera-Bailón et al., 2013; López-López et al., 2017).

All animals submitted to the high-intensity acoustic stimulation protocol were evaluated according to the severity index (SI) described by Garcia-Cairasco et al. (1996). The hamsters corresponding to the control group exhibited normal hearing with positive Preyer’s reflex and absence of seizures with a SI score of 0. The GASH/Sal animals corresponding to the high-intensity acoustic stimulation group (GASH/Sal Stim) exhibited all the consecutive phases of the audiogenic seizures with generalized tonic–clonic seizures and clonic spasms, and hence reached the maximum SI (scores of 8). These GASH/Sal animals underwent audiogenic seizures that are very stable and specifically dependent upon the high intensity acoustic stimulation with a duration as short as 10 s (Sánchez-Benito et al., 2017). After acoustic stimulation, the seizure appeared within seconds and lasted for approximately 5 min (Sánchez-Benito et al., 2017).

All experimental procedures and protocols were performed in accordance with the guidelines of EU (Directive 2010/63/UE) for the care and use of laboratory animals and approved by the Bioethics Committee of the USAL (approval number 300). All efforts were made to minimize the number of animals and their suffering. After weaning, the animals were separated from the colony and maintained in Eurostandard Type III cages (Tecniplast, Italy), containing up to 4 individuals, with Lignocel bedding (Rettenmaier Iberica). The animals were maintained in an acoustically controlled environment under 14/10 light/dark cycles at constant room temperature (22–24°C) and *ad libitum* access to food (Teckad Global 2918 irradiated diet) and water. The absence of seizures before starting the experiments was ensured by the strict control of housing conditions and handling.

### RNA Isolation

Tissue samples for each set of experiments were obtained and processed in parallel for both animal groups, and were performed at the same time of the day (early in the morning). The euthanasia and tissue collection for gene expression analysis were set 60 min after the high acoustic stimulation as established previously by López-López et al. (2017). For tissue sampling, all animals were deeply anesthetized under gas anesthesia (2.5% isoflurane), the brains were removed quickly after decapitation and the IC was isolated, surgically removed, and placed in QIAzol Lysis Reagent (#79306, QIAgen). Then, the samples containing the IC tissue were homogenized using TissueLyser II instrument (#85300, QIAgen), according to the manufacturer’s instructions. After separating the different phases, the aqueous phase was used to extract RNA following the instructions of the RNeasy Mini Kit (#74104, QIAgen). RNA concentrations and quality were assessed using an Agilent 2100 Bioanalyzer to assess the integrity of the 18S and 28S rRNA bands, as well as an RNA integrity number (RIN) > 8.0, with 0 corresponding to fully degraded RNA and 10 corresponding to intact RNA, were used for data analysis (López-López et al., 2017).

### RNA-Seq and Data Analysis

To generate cDNA libraries of the IC of GASH/Sal and control hamsters, RNA samples were pooled from 6 GASH/Sal and 6 control hamsters after loud sound stimulation, mixing 1 μg of each sample. The two-cDNA libraries were prepared using 3 μg of total RNA of each animal group, and the TruSeq RNA Sample prep kit v2 (Illumina), adding a capture step of RNA polyA to eliminate rRNA. Subsequently, both cDNA libraries were sequenced on a Genome Analyzer IIx (Genome Analyzer IIx, Illumina) in the single read format (1 × 75 bp) at the Cenit Support Systems laboratory (Salamanca, Spain).

The purity criteria of samples for cDNA library construction were determined using the Illumina sequencer software FASTQC (Andrews, 2010) to test the quality of the samples and to select the most suitable parameters for alignment. All our samples successfully passed this quality control step (Phred, 0–40). Then, the reads were aligned and counted on the reference genome of Syrian hamster MesAur1.0 (GCA_000349665.1) (Tchitchek et al., 2014) using the STAR software (Dobin et al., 2013). Lastly, a differential expression analysis between the groups of samples (GASH/Sal *vs.* control) was performed using the EdgeR statistical package (Robinson et al., 2010). Genes with a rate of change (log Fold-Change) higher than 1.5 (over-expressed) or lower than 1.5 (under-expressed) [|logFC| ≥ 1.5] and with a count higher than 40 reads were selected for further analysis in this study.

### Quantitative Reverse Transcription Real-Time PCR (RT-qPCR)

RT-qPCR analysis was used to confirm a set of 27 gene expression changes observed in the RNA-seq. For our RT-qPCR analysis, we used an aliquot of the same RNA samples as those used for the RNA-seq experiment, and additionally, new RNA samples from 6 GASH/Sal with sound-induced seizures and 6 sound-stimulated controls were also used. We also used RNA samples from the naïve GASH/Sal group (*n* = 6), corresponding to seizure-prone animals that did not develop any audiogenic seizure.

For reverse-transcription, we followed the protocol routinely used in our laboratory (López-López et al., 2017). Briefly, total RNA (2 μg) was mixed with oligo-dT and random hexamer primers for reverse-transcription into cDNA using the First Strand cDNA Synthesis Kit (K1621, Promega Corporation, Madison, WI, United States). In all cases, a reverse transcriptase negative control was used to test genomic DNA contamination.

Subsequently, quantitative qPCR was performed using the SYBR Green method with a 2 × Master Mix (#4367659, Applied Biosystems). Each reaction contained 10 μL of Master Mix, 0.4 μL of each pair of primers, 3 μL of each cDNA sample in a different serial cDNA quantity for each gene, and MilliQ water (RNA free) up to 20 μL. The amplification reaction was performed in the QuantStudio 7 Flex Real-Time PCR System (Applied Biosystems) under the following conditions: 10 min at 95°C followed by 40 cycles of 15 s at 95°C and 30 s at 60°C depending on each pair of primers. RT-qPCR experiments were performed in replicates of 6 to 7 samples and conducted in triplicate for each gene product examined. The list of primers used is provided in Table 1.

**TABLE 1:** Oligonucleotide primers used for RT-qPCR, indicating the location of each primer in the corresponding Ensembl sequences of the Syrian hamster^(*a*)^.

**Gen target**	**ID transcript ensembl *Mesocricetus auratus*^*a*^**	**Primer forward**	**Primer reverse**	**Size of products**	**E^*b*^**
*Egr1*	*ENSMAUG00000007358*	CAGC(A/G)GCGC(T/C)TTCAATCCTC	GTGGTCAGGTGCTCGTAGGG	60	2.04
*Egr2*	*ENSMAUG00000021143*	AGGCCCTTGGATCTCCCATA	CAGCTGGCACCAGGGTACTG	162	2.00
*Egr3*	*ENSMAUG00000000747*	CCACAAGCCCTTCCAGTGTC	GTGCGGATGTGAGTGGTGAG	75	1.98
*Ttr*	*ENSMAUG00000011770*	GCCTCGCTGGACTG(G/A)(C/T)ATTT(A/G)	TCGGACAGCATCCAGGACTT	85	2.00
*Rxfp2*	*ENSMAUG00000017558*	AAGCTGTGCCAAAGGTTTCTA	TTGCTGAAAACTTTGACTGGAA	88	1.99
*C6*	*ENSMAUG00000007780*	CTGTGTCCTTGGAGACTACGG	GTCACCAGAGGTTCTGTGCAT	126	1.98
*Atp2a3*	*ENSMAUG00000005976*	TGTGTGGCTGTATGGGTCAT	GCCACGGCAATCTTGAAGTA	92	1.98
*Rab29*	*ENSMAUG00000017388*	TTGCTCTGAAGGTTCTCCAGT	GGCTGTTGCTGAAAGTAGTGG	168	2.06
*Gm12695*	*ENSMAUG00000020018*	GGTCTCCCCAGAAGAAAGTCT	AGGCTGGAGTTCAATGGGTA	162	1.94
*Grin2c*	*ENSMAUG00000019504*	GTTCAGCCGTTGGCCTCTAT	ACCCAGATCACACCAGACCT	90	1.90
*Renbp*	*ENSMAUG00000019504*	CGAGCACAAGTCATTGACAAA	ATCATGGCTTCACTGTGTGG	158	2.09
*Slc6a4*	*ENSMAUG00000012677*	GCGGTACTGGATGAGTTTCC	TCTATGAGTGCCACCGTGAG	176	1.98
*Mmp3*	*ENSMAUG00000011335*	CCGTGATACCCACCAAATCT	GGGCCAAAATGAAGAGATCA	92	1.93
*Atf3*	*ENSMAUG00000011335*	GGCAACTGGGGAGTCCTTAT	GAGACGAAGGATGCTCTTGC	120	1.98
*Gadd45g*	*ENSMAUG00000011335*	TTGCTGTTCTTGGATCGTACA	GACTTTGGCGGACTCGTAGA	168	1.89
*Slc13a4*	*ENSMAUG00000018591*	AGGGGATAGAGCCCATCATC	GCTGACAAACTCCGTGACAA	165	2.07
*Npas4*	*ENSMAUG00000016391*	GGCTACATTCCTTTCCGATG	CTACAAAGTCACCGCAGCAC	149	2.01
*Wdr38*	*ENSMAUG00000005054*	AGCTTCAGCCCTGACTCAAA	GAGGCTGAGTAGCACAAGCA	65	2.07
*Ogn*	*ENSMAUG00000011988*	ACCATTGCCAAAGGAATCAG	GTTCTTCTAACAGAGACAGTTTTGAA	170	2.08
*Sucnr1*	*ENSMAUG00000019748*	CAGTCTGTGCCTGACTTTGC	ACAGAGAAGATCGCCACCAC	164	1.95
*Fos*	*ENSMAUG00000019419*	CAGCTCGCACCAGTGTCTAC	ACTTCCGGAAAACATCATGG	76	2.00
*Kcns1*	*ENSMAUG00000000788*	CGCTTGTGCGATGATTATGA	ACTCGCCGCTCCAGATAG	100	2.02
*Junb*	*ENSMAUG00000004233*	GCAGCTACTTTTCGGGTCAG	TTCATCTTGTGCAGGTCGTC	200	1.97
*Kcnj13*	*ENSMAUG00000010435*	TCAAAGATACCGGAGGATGG	CAAAGACAAGCCAGTGGACA	178	2.07
*Cd163*	*ENSMAUG00000000759*	AGAAGAGAAGCGGAGGGTTC	ACCAGGACAAACTCCAGACG	163	2.03
*Fosb*	*ENSMAUG00000000999*	AGAAGAGAAGCGGAGGGTTC	ACCAGGACAAACTCCAGACG	182	2.09
*Slc28a1*	*ENSMAUG00000015388*	TTAATTGCTGCCTCCGTAATG	GAACTTGGACTCCTCCACCTC	84	1.94
*Actb*	*ENSMAUG00000008763*	AGCCATGTACGTAGCCATCC	ACCCTCATAGATGGGCACAG	105	2.03

*qPCR primer efficiency (E^*b*^) was calculated according to the following equation: *E* = 10^(–*1*/*slope*)^.*

A standard curve was made to verify the efficiency (E) of the primers of the target and reference genes and it was constructed by serial dilutions of cDNA isolated: 80, 40, 20, 10, 5, 2.5 and 1.25 ng/μl. Data showed that all genes used in this work were expressed at a high level and investigated transcripts showed high linearity (*R*^2^ > 0.95). Real-time PCR efficiencies of one cycle in the exponential phase were calculated according to the equation *E* = 10^[–1/slope]^. High PCR efficiency rates were shown to occur in the investigated range of nanogram cDNA input, and all genes produced approximately identical slopes (Table 1).

To decide which was the most stable gene as an endogenous reference for RT-qPCR data normalization two candidates [β-actin (*Actb*) and glyceraldehyde 3-phosphate dehydrogenase (*Gapdh*)] were selected and their expression was measured by NormFinder software (Andersen et al., 2004) that calculate intra- and intergroup variations in gene expression. Thus, the mean threshold cycle (Ct) value and primer efficiency value of *Actb* were used for data normalization.

The comparative Ct method was used for quantitative data analysis (Schmittgen and Livak, 2008). After removing outliers (Burns et al., 2005), the relative gene expression value (FC) of each transcript was calculated according to the formula 2^−(Δ*Ct*^“^*condition*1^”^–^^Δ*Ct*^ “^*condition*2^”), where in “condition 1” corresponds to the experimental sample, “condition 2” to the sample from the control animal, and the ΔCt of each “condition” is Ct_“experimental gene”_ - _Ct “endogenous gene”_ (Schmittgen and Livak, 2008). The standard error of each relative gene expression value was calculated as a measure of data variation. Significant differences in qPCR results were determined using the Student’s *t*-test, and the results were considered significant when *p < 0. 05, **p < 0. 01, and ***p < 0. 001. The data were plotted using GraphPad Prism (version 6.05).

The confirmed differentially expressed genes by RT-qPCR were functionally classified using biological databases available on web platforms such as The PANTHER (Protein Analysis Through Evolutionary Relationships) Classification System,^1^ STRING 10.0,^2^ KEGG (Kyoto Encyclopedia of Genes and Genomes)^3^ and the Consortium of Genetic Ontology.^4^ Both Fisher’s exact test and the hyper-geometric test were used to identify significantly overrepresented functional categories, with at least 3 annotated genes, at *p-*value < 0.05.

### Metabolomic Studies

For metabolomics analysis, we evaluated overrepresentation in metabolic pathways using a hypergeometric distribution test. Since the metabolome of the Syrian hamster has not been described yet, the identifiers were converted into their human equivalent using the databases KEGG, Reactome Pathway Database (Reactome)^5^ and the Human Gene Database (GeneCards).^6^

To correct false positives in this analysis, a False Discovery Rate was applied using the Benjamini-Hochberg’s method (Benjamini and Hochberg, 1995). Interactions between unrelated metabolic pathways have been considered to maximize the scope of analysis. An enrichment network representing gene sets in grouped nodes was performed in the study set (Reimand et al., 2019). In addition, an overrepresentation analysis of the total data was performed to identify any priority pathway in the epileptogenic focus. To determine the gene expression level groups, k-means clustering was performed (MacQueen, 1967). This clustering makes it possible to establish relationships between distant genes based on the assumption that related genes will be expressed similarly. Both metabolomic analyses were exclusively performed in the differentially expressed genes of interest that were confirmed using the RT-qPCR approach.

## Results

### Analysis and Comparison of the IC Transcriptomes

**FIGURE 1:**
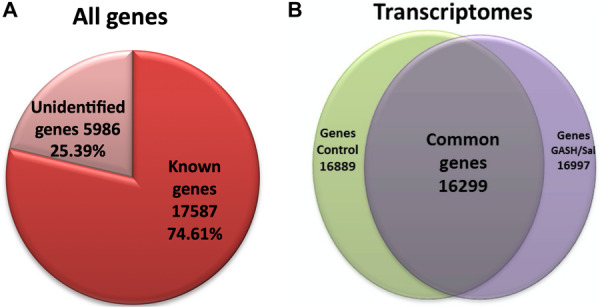
Number of genes detected and expressed in the IC transcriptomes of GASH/Sal and control hamsters after high acoustic stimulation. **(A)** Total number of detected genes in the RNA-Seq experiment. **(B)** Total known genes that were expressed in both transcriptomes. Notice that 16299 correspond to genes that were expressed in both experimental groups. 16889 were those genes expressed in the control IC, out of which 590 genes did not meet the selection criteria, and hence were not considered in this study. 16997 correspond to the genes expressed in the IC of the GASH/Sal, out of which 698 genes did not meet the selection criteria and were excluded.

After the high-intensity acoustic stimulation, the transcriptomes of the IC in the GASH/Sal and Syrian control hamsters were obtained and analyzed using the Syrian hamster as reference genome (Tchitchek et al., 2014). A total of 23573 genes were detected in both transcriptomes; out of which 17587 genes were identified as known groups of genes, and the other 5986 genes matched with transcripts from unidentified genes (Figure 1A). Out of the total 17587 transcripts from known genes, 16299 were common genes identified in both control and GASH/Sal hamsters (Figure 1B). Bioinformatics analysis of the IC transcriptomes in both animal groups showed 23–25 million short-insert Illumina reads, with 93% average mappings in a length of 75 bp, 7–8% splice sites and a 45–55% GC content (Table 2). No anomaly or deviation was detected in any quality control for all samples. The sequences corresponding to the IC transcriptomes of GASH/Sal and control hamsters after high-intensity acoustic stimulation were made available to the scientific community by depositing them in NCBI (Supplementary Figure S1).

**TABLE 2:** Data from the alignments of RNA-Seq reads of the epileptogenic focus in GASH/Sal and control hamsters after loud sound stimulation.

**Sample**	**Uniquely mapped reads number**	**Uniquely mapped reads (%)**	**Average mapped length**	**Mismatch rate per base (%)**	**Deletion rate per base (%)**	**Deletion average length**	**Insertion rate per base**	**Insertion average length**	**Splice site (%)**	**CG content (%)**
GASH/Sal	25955861	93.20	75.64	0.31	0.01	1.58	0.00%	1.38	7.91	45
Control	23412284	93.40	75.65	0.31	0.01	1.58	0.00%	1.4	8.01	55

On the other hand, the gene expression analysis of the IC transcriptomes in the GASH/Sal and control hamsters after loud sound stimulation provided a list of 16299 commonly expressed genes (Figure 1).

### Top Differentially Expressed Genes Selected With Specific Criteria

Of the total 16299 commonly expressed genes, 36 genes were specifically selected using a cut off threshold of (|logFC| ≥ 1.5), normalized gene expression value above 10 counts per million (CPM) and a reads overlapping (COUNTS) greater than 40.

**FIGURE 2:**
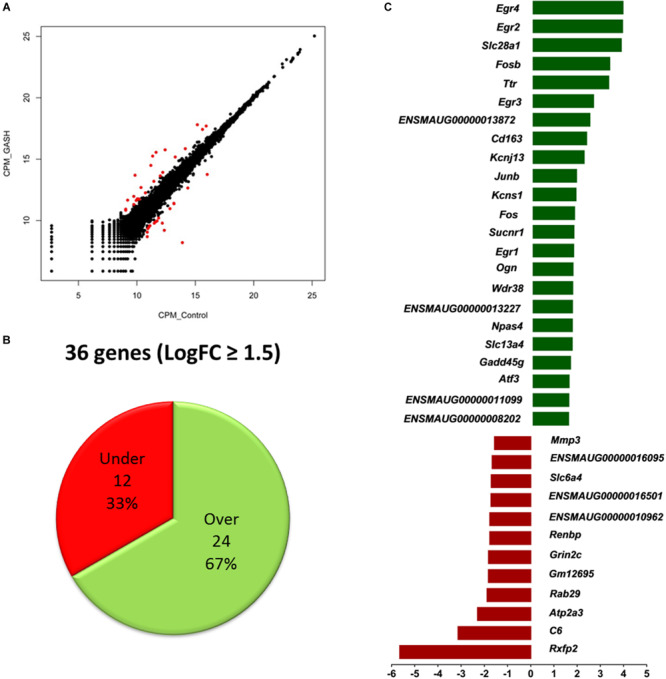
Top differentially expressed genes selected by the criteria of |LogFC| ≥ 1.5, CPM > 10 and COUNTS > 40 when comparing the IC transcriptomes of the GASH/Sal hamster and the Syrian control hamster after high-intensity acoustic stimulation. **(A)** Scatterplot matrix of the expression value (measured as counts per million mapped reads, CPM) of each gene in GASH/Sal *vs*. control samples. Genes with significant differential expression are represented in red. **(B)** Number of differentially expressed genes. **(C)** Identification of differentially expressed genes over- (in green) and underexpressed (in red).

Of these 36 genes, 24 genes were found to have significantly increased mRNA expression levels (at least 1.5-fold), whereas the mRNA expression levels of 12 genes was significantly decreased (at least 1.5-fold) when comparing the IC of the GASH/Sal and the control hamsters. Moreover, of 36 differentially expressed genes, 29 genes were known and 7 were unknown genes, with undefined sequences (Figure 2 and Table 3).

**TABLE 3:** Analysis of RNA-seq data of the differentially expressed genes with |logFC| ≥ 1.5, CPM > 10 and COUNTS > 40 in the IC transcriptome of GASH/Sal in comparison with the IC transcriptome of the Syrian control, both after acoustic stimulation.

**Symbol**	**CPM_Control**	**CPM_GASH**	**FC**	**LogFC_CPM**	**Full name**
*Egr4*	3197.37	48350.78	15.15	3.92	Early Growth Response 4
*Egr2*	2611.30	39070.74	15.00	3.91	Early Growth Response 2
*Slc28a1*	918.19	13240.31	14.52	3.86	Solute Carrier Family 28 Member 1
*Fosb*	5476.56	55621.13	10.17	3.35	FosB Proto-Oncogene, AP-1 Transcription Factor Subunit
*Ttr*	2350.82	23111.44	9.86	3.30	Transthyretin
*Egr3*	36994.47	229400.22	6.20	2.63	Early Growth Response 3
*ENSMAUG00000013872*	592.59	3250.97	5.55	2.47	
*Cd163*	1308.90	6620.16	5.08	2.35	CD163 Molecule
*Kcnj13*	853.07	4019.38	4.75	2.25	Potassium Voltage-Gated Channel Subfamily J Member 13
*Kcns1*	527.47	1950.58	3.74	1.90	Potassium Voltage-Gated Channel Modifier Subfamily S Member 1
*Junb*	9969.82	37120.16	3.73	1.90	Junb Proto-Oncogene, AP-1 Transcription Factor Subunit
*Fos*	50278.88	176025.21	3.50	1.81	Fos Proto-Oncogene, AP-1 Transcription Factor Subunit
*Egr1*	62586.49	213145.37	3.41	1.77	Early Growth Response 1
*Ogn*	2806.66	9398.26	3.36	1.75	Osteoglycin
*Wdr38*	1048.43	3487.40	3.35	1.74	WD Repeat Domain 38
*Npas4*	18109.77	59285.86	3.27	1.71	Neuronal PAS Domain 4
*Slc13a4*	4760.25	15545.54	3.27	1.71	Solute Carrier Family 13 Member 4
*ENSMAUG00000013227*	983.31	3191.86	3.27	1.71	
*Sucnr1*	527.47	1832.36	3.13	1.64	Succinate Receptor 1
*Gadd45g*	4109.05	12767.44	3.11	1.64	Growth Arrest And DNA Damage Inducible Gamma
*Atf3*	853.07	2541.67	3.00	1.59	Activating Transcription Factor 3
*ENSMAUG00000011099*	1178.67	3428.29	2.92	1.55	
*ENSMAUG00000008202*	1699.62	4906.01	2.90	1.53	
*Mmp3*	2090.34	709.30	0.34	−1.56	Matrix Metallopeptidase 3
*ENSMAUG00000016095*	3718.33	1182.17	0.32	−1.65	
*Slc6a4*	3067.14	945.74	0.31	−1.70	Solute Carrier Family 6 Member 4
*ENSMAUG00000016501*	21040.16	6442.83	0.31	−1.71	
*Renbp*	3392.73	1004.85	0.30	−1.76	Renin Binding Protein
*ENSMAUG00000010962*	9188.38	2718.99	0.30	−1.77	
*Grin2c*	9253.50	2659.88	0.29	−1.81	Glutamate Ionotropic Receptor NMDA Type Subunit 2C
*Rab29*	7299.91	2009.69	0.28	−1.87	RAB29, Member RAS Oncogene Family
*Atp2a3*	66493.67	13831.40	0.21	−2.28	ATPase Sarcoplasmic/Endoplasmic Reticulum Ca2+ Transporting 3
*C6*	5216.08	591.09	0.11	−3.13	Complement C6
*Rxfp2*	15244.51	295.54	0.02	−5.69	Relaxin Family Peptide Receptor 2

### Validation by Quantitative Reverse Transcription Real-Time PCR (RT-qPCR)

**FIGURE 3:**
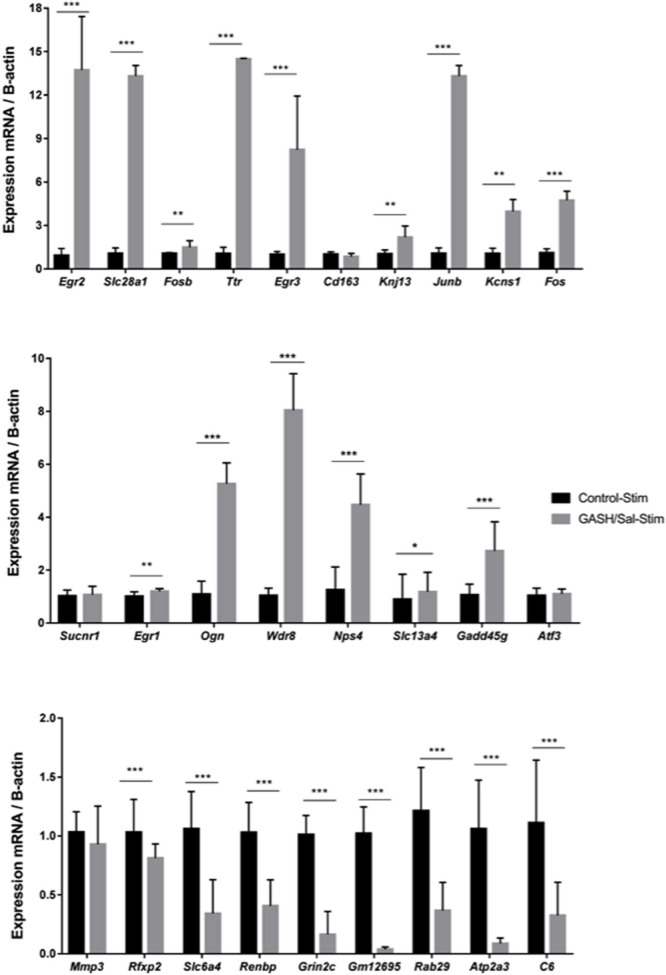
mRNA expression levels of IC genes in GASH/Sal and control animals after high-intensity acoustic stimulation. Histograms show mRNA expression levels of 27 genes selected from the comparative transcriptome analysis shown in Figure 2. Asterisks indicate statistically significant differences in expression of each gene in the IC of GASH/Sal animals as compared to controls. Significance was set at *p < 0.05, **p < 0.01, ***p < 0.001. Error bars indicate hemi-standard deviation (SD).

We performed RT-qPCRs to validate a set of differentially expressed genes (27) found in the comparative RNA transcriptome analysis of the GASH/Sal and control inferior colliculi. The genes tested for RT-qPCR confirmation were selected based on the two specific criteria (|logFC| ≥ 1.5, CPM > 10 and COUNTS > 40), the ontological categories and/or their potential roles in epileptogenic events. As shown in Figure 3, these analyses confirmed that *Egr2, Slc28a1, Fosb, Ttr* (Transthyretin), *Egr3, Kcnj13, Junb, Kcns1, Fos, Egr1*, *Ogn* (Osteoglycin), *Wdr38 (WD Repeat* domain 38), *Slc13a4, Npas4* and *Gadd45g* (Growth arrest and DNA damage inducible gamma) were overexpressed in the GASH/Sal *vs.* control hamsters after high acoustic stimulation. In addition, the genes *Rfxp2* (Relaxin family peptide receptor 2), *Slc6a4, Renbp* (Renin binding protein*), Grin2c* (Glutamate ionotropic receptor NMDA type subunit 2C), *Gm12695* (Chromosome unknown C1orf87 homolog), *Rab29* (RAB29, member RAS oncogene family), *Atp2a3* (ATPase sarcoplasmic/endoplasmic reticulum Ca^2+^ transporting 3) and *C6* (Complement C6) were significantly down regulated in GASH/Sal animals, when compared with control hamsters. The genes *Sucnr1* (Succinate receptor 1), *Cd163* (CD163 Molecule), *Atf3* (Activating transcription factor 3) and *Mmp3* (Matrix metallopeptidase 3) showed no significant difference between the two transcriptomes, and the *Egr4* gene could not be analyzed because this transcript is associated with several mRNA products.

**FIGURE 4:**
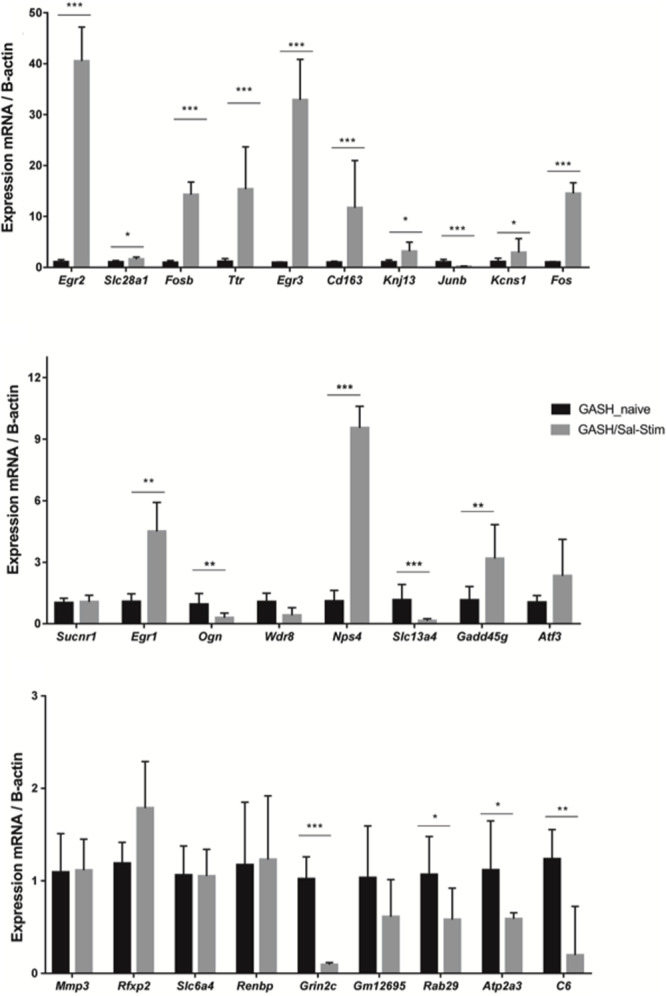
mRNA expression levels of IC genes in GASH/Sal under-free seizure conditions (GASH/Sal naïve) and GASH/Sal animals with seizures after high-intensity acoustic stimulation (GASH/Sal-Stim). Histograms show mRNA expression levels of 27 genes selected from the comparative transcriptome analysis shown in Figure 2. Asterisks indicate statistically significant differences in expression of each gene in the IC of GASH/Sal animals with audiogenic seizure as compared to GASH/Sal naïve. Significance was set at *p < 0.05, **p < 0.01, ***p < 0.001. Error bars indicate hemi-standard deviation (SD).

Using the RT-qPCR approach, we further analyzed the differential gene expression between the GASH/Sal under free-seizure conditions and the GASH/Sal with sound-induced seizures to determine the gene expression changes due to the effects of having an audiogenic seizure. As shown in Figure 4, these analyses confirmed that *Egr2, Slc28a1, Fosb, Ttr*, *Egr3, Kcnj13, Kcns1, Fos, Egr1*, *Ogn, Npas4* and *Gadd45g* were overexpressed in the GASH/Sal with audiogenic seizures. In addition, the genes *Junb, Slc13a4, Grin2c, Rab29*, *Atp2a3* and *C6* were significantly down regulated in GASH/Sal under free-seizure conditions, when compared to GASH/Sal with sound induced-seizures. On the other hand, the genes *Wdr38, Sucnr1, Cd163, Atf3, Mmp3, Rfxp2, Slc6a4, Renbp and Gm12695*, showed no significant difference between the two experimental conditions.

Finally, we did not evaluate some genes such as ENSMAUG00000010962, ENSMAUG00000016501, ENSMAU G00000016095, ENSMAUG00000008202, ENSMAUG000000 11099, ENSMAUG00000013227 and ENSMAUG00000013 872 because they had no products or defined sequences.

### Functional Association Networks and Gene Ontology Analysis of the Top Differentially Expressed Genes

**FIGURE 5:**
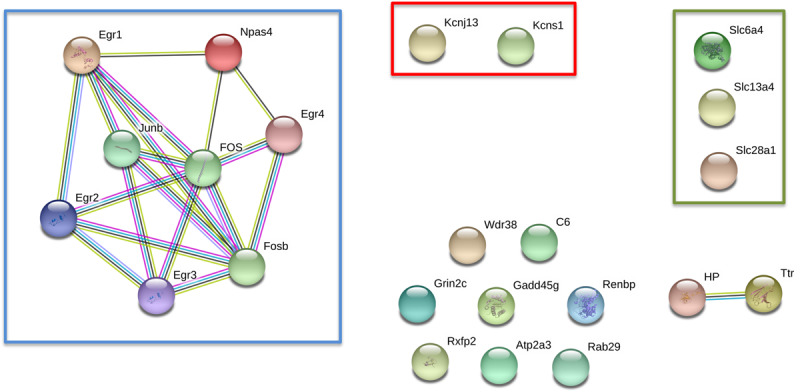
Network-Based Integration of 24 differentially expressed genes in the IC of GASH/Sal *vs.* control hamsters after high-intensity acoustic stimulation. In red box: genes encoding potassium voltage-gated channels; green box: genes encoding solute carrier proteins, and blue box: molecular network.

To better understand the possible functional association networks resulting from the altered mRNA expression in the epileptogenic nucleus of the GASH/Sal, we searched for the available protein-protein interactions of the 24 confirmed differentially expressed genes using the software STRING. The STRING database contains information on known and predicted, direct physical, and indirect functional protein-protein interactions. This analysis showed interactions of early growth response genes (***Egr1***-***4***), the genes encoding proto-oncogene AP-1 transcription factor subunit (***Fos, FosB, Junb***) and the gene encoding for the neuronal PAS domain protein 4 (***Npas4***) (Figure 5). The level of trust of the associations is represented by the thickness and number of lines with a *p*-value < 0.05 (interaction score in STRING database > 0.95). Based on this network analysis, we generated the following interaction networks: 13.7% of the genes were related to early growth response; 10.3% were transcriptional factors such as ***Fos, FosB*** and ***Junb***; 10.3% encoded solute carrier proteins (***Slc28a1***, ***Slc13a4*** and ***Slc6a4***); and 6.8% encoded potassium voltage-gated channels (***Kcnj13***, ***Knsc1***), among others (Table 3).

**FIGURE 6:**
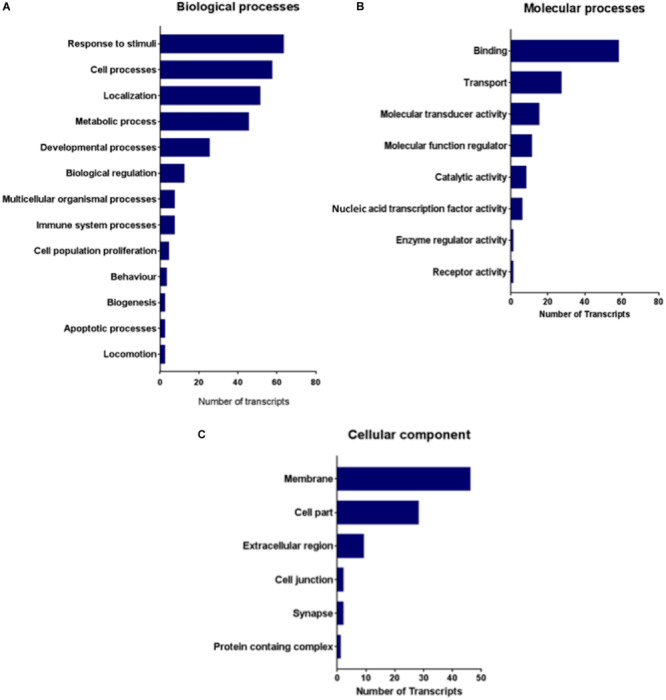
Represented gene ontology terms of 24 differentially expressed genes in the IC comparative transcriptomes of GASH/Sal *vs.* control hamsters after high-intensity acoustic stimulation. **(A)** Biological processes, **(B)** molecular processes, and **(C)** cellular components.

To further analyze the functionality of the networks, we carried out a PANTHER analysis of the 24 differentially expressed genes to determine which functional Gene Ontology (GO) categories (molecular process, biological process and cellular component) were highly represented (Figure 6).

### Metabolomics Analysis

Metabolomics analysis using the 16299 differentially expressed genes in the epileptogenic focus, showed no significant difference, which implies an overall expression balance between metabolic pathways. On the other hand, the 36 differentially expressed genes were studied, and when performing their metabolomic analysis, 318 pathways contained at least one of those genes. Applying the criterion to confer a metabolic pathway with a *p* < 0.01 as significant, we found that the most affected routes by the overexpression of these genes were the interleukins- 4 and -13 (IL-4 and IL-13) signaling (10.3180/R-HSA-6785807.1), and the transporters of both nucleosides and free bases in the plasma membrane (10.3180/REACT_1206.3) (Table 4).

**TABLE 4:** Metabolic pathways analyzed using the KEEG, REACTOME and GeneCards databases.

**Pathway**	**Entities found**	**Entities total**	**Interactions found**	**Interactions**	***p*-valor**	**Reactions**	**Reactions total**
Transport of nucleosides and free bases in the plasma membrane	21	3	0	7	1.88E–03	2	16
Signaling IL-4 Il-13	7	211	0	138	1.66E–05	2	46

*This analysis identifies significantly overrepresented functional annotations.*

**FIGURE 7:**
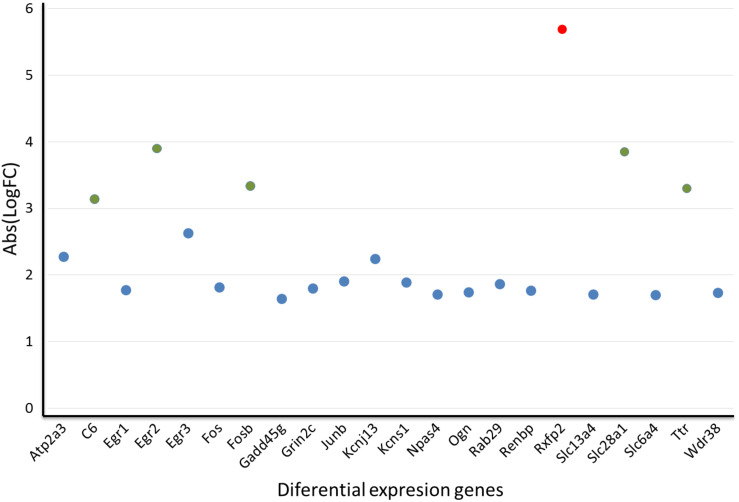
Gene distribution in each metabolic cluster of 24 differentially expressed genes in the IC of sound-stimulated GASH/Sal hamsters in relation to their controls. Red circle: In cluster 1, the *Rxfp2* stands out from the other genes as an outlier; Blue circle: cluster 2 includes all genes studied with no significant differences; Green circle: cluster 3 groups five genes with significant differences.

Of the top differentially expressed genes, 21 of them have a human homolog described in the metabolome. These 21 genes were clustered according to their gene expression levels by k-means clustering (MacQueen, 1967), which resulted in 3 clusters (Figure 7). In cluster 1, the *Rxfp2* gene stands out from the other study genes as a single-member cluster (red circle, Figure 7). Cluster 2 includes all study genes with no significant differences between them and expressed at standard levels (*Atp2a3, Egr1, Egr3, Fos, Gadd45g, Grin2c, Junb, Kcnj13, Kcns1, Npas4, Ogn, Rab29, Renbp, Slc13a4, Slc6a4, Wdr38*; as shown in blue circles in the Figure 7). Lastly, cluster 3 groups five genes (*C6, Egr2, Fosb, Slc28a1* and *Ttr*) with significant differences from the two previous groups (green circles, Figure 7). Overrepresentation analysis of cluster 3 (which is of greater interest in our study) showed that the route with the lowest *p*-value corresponds to the transport of nucleosides and free bases in the plasmatic membrane (*Slc28a1*). Furthermore, cluster 3 has genes corresponding to metabolic pathways, a finding that reinforces the results from our metabolomics analyses and highlights interleukins IL-4 and IL-13 signaling and nucleoside transport as possible damaged or altered routes in our model.

## Discussion

Epilepsy is a neurological disorder with a high epidemiological impact worldwide. In this context, epilepsy research on experimental animal models plays a critical role in determining the cellular and molecular factors underlying ictogenesis and epileptogenesis, searching for parallel factors with human epilepsies. In the present study, RNA-seq was used to identify changes in gene expression in the epileptogenic focus (namely the IC) between GASH/Sal and control hamsters after high-intensity sound stimulation.

The RNA-Seq approach is a sensitive and accurate method for the quantification of gene expression levels. Despite its reliability, the RNA-Seq data exhibits some variations due to normalization and differences in probes which make necessary to be validated by RT-qPCR (Wang et al., 2009). Accordingly, our RNA-Seq results were confirmed by RT-qPCR, showing 24 differentially expressed (known) genes between the IC of GASH/Sal and control animals. Moreover, to understand the possible functional associations and the biological relevance of these genes, we searched for significantly overrepresented biological processes or molecular functions in several ontological processes such as positive regulation of transcription through RNA polymerase II promoter, transport, voltage-gated ion channel activity and transcription factor complex.

### Methodological Discussion

In the present study, the altered gene expression in the IC of the GASH/Sal with sound-induced seizures was determined by a comparative transcriptome analysis using RNA-seq. This allowed us to interpret in a global and comprehensive manner the functional elements of the genome and reveal differences in gene expression between cells and tissue from different sources. The key objectives of the transcriptome are: to catalog the transcripts, including mRNA, non-coding RNA and small RNA and to determine transcriptional structures and quantify expression levels (Wang et al., 2009).

In our study, we assessed the gene expression levels of the IC in GASH/Sal with sound-induced seizures and control hamsters that were subjected to the same acoustic stimulation protocol. For such purpose, we used an interval of 60 min between the high-intensity acoustic stimulation and the animal euthanasia for the extraction of the IC tissue samples. Subsequently, we identified the immediate-early genes that activate metabolic processes induced by response to a stimulus (Vaudry, 2002; Fowler et al., 2011), as suggested in studies with early growth response genes (López-López et al., 2017). In addition, our bioinformatics evaluation of the RNA-seq pool of IC transcripts in GASH/Sal with audiogenic seizures *vs*. sound-stimulated control hamsters followed a restrictive criteria that included the |logFC| ≥ 1.5 ranges and a normalized expression value higher than 10 counts to select differentially expressed immediate-early genes. Lastly, a set of transcripts selected as differentially expressed were confirmed by RT-qPCR in order to validate the differentially expressed genes that were previously identified in the IC transcriptomes of GASH/Sal and control hamsters after high-intensity acoustic stimulation.

The RT-qPCR approach quantifies the expression levels of a given gene by fluorescence, which is directly proportional to the amplification of the target cDNA. The analysis of these results requires determining the value of baseline fluorescence or background noise, which does not correspond to the amplification of the sample. This value is calculated by automated analysis of the amplification graph. The qPCR machine provides the threshold value, which refers to the increase in fluorescence that is considered significant with respect to the baseline value. Ultimately, Ct is defined as the cycle at which the fluorescence exceeds the threshold value. The results are normalized and analyzed based on the values of Ct of the different samples for each gene of interest. Data normalization in quantitative experiments, such as qPCR, requires using constitutive genes as calibrators. These constitutive or reference genes show stable expression, regardless of cell type or treatment applied (Chapman and Waldenström, 2015). In this study, *Actb* was used as the reference gene because it was the most stable gene according to the expression level measured by NormFinder software in the intra- and intergroup analyses (Andersen et al., 2004),. Moreover, it has been previously reported that the *Gapdh* gene is overexpressed in the Syrian hamster (McCann et al., 2017). Reference genes make it possible to normalize the amount of cDNA used in each reaction. The results were analyzed using the 2^–ΔΔ*CT*^ method, which quantifies relative changes in gene expression. These changes were expressed as FC values, which have been normalized using the reference gene and in relation to the control condition (Schmittgen and Livak, 2008).

Differences between RT-qPCR and RNA-seq experiments used for selecting the commonly expression genes in the comparative analysis of both transcriptomes occur for several reasons, including the fact that different probes are used for the RT-qPCR and RNA-seq experiments, differences in the methods for normalization of expression data and possible false-positive expression changes (Costa et al., 2013).

### Molecular Networks of Differentially Expressed Genes

In the molecular network analysis, the EGR gene family was identified as the main axis of gene interactions. These genes encode a family of zinc-finger proteins, which bind to DNA, RNA, or proteins (Crosby et al., 1991; O’Donovan et al., 1999). *Egr1, Egr2*, and *Egr3* are immediate-early genes whose transcription can be rapidly and transiently induced by a broad range of cellular stimuli, including environmental, physiological, and pathological stimuli (Beckmann and Wilce, 1997; López-López et al., 2017). In this network, we also identified *Fosb*, *Junb* and *Fos* genes that were overexpressed in the GASH/Sal model in relation to the respective controls. This is a key alteration because these genes encode transcription factors involved in several biological processes, including cell proliferation, differentiation, apoptosis, and inflammation (Hess, 2004). Another overexpressed gene that belongs to this network is *Npas4*, a neuron-specific transcriptional factor critical for activity-dependent regulation of GABAergic synapse development *in vitro* through BDNF expression (Lin et al., 2008). *Npas4* gene is directly involved in activity-dependent gene expression control and regulation of long-lasting brain functions, such as memory formation, adaptation, and synaptic plasticity (Lin et al., 2008; Ye et al., 2016). Lastly, *Egr* genes have been also reported in previous studies on epilepsy in the GASH/Sal hamster (López-López et al., 2017), in polycarpic-induced rat models (Lösing et al., 2017), in patients with refractory epilepsy (Liu et al., 2016), in the IC of the DBA/2J mice after induced audiogenic seizures (Li and Hu, 2005) and in an animal model of temporal lobe Epilepsy (Grabenstatter et al., 2014). Similarly, the genes *Npas4, Junb, Fos* and *Fosb* have also been related to epileptiform processes in animal models and epileptogenic tissue samples (Elliott and Gall, 2000; Beaumont et al., 2012; Liu et al., 2016; Lösing et al., 2017).

Overall, the GO analysis of this molecular network demonstrated that several GO terms identified in this study have been related to epilepsy in previous studies on refractory epilepsy of the mesial temporal lobe (Bando et al., 2011) and in rat models of febrile seizures (Wang et al., 2014). In addition, this molecular network was also identified in conditional serum response factor (SRF) knockout mice and mouse pilocarpine epilepsy models (Kuzniewska et al., 2016; Lösing et al., 2017). Thus, overexpression of transcriptional factors, growth factors and *Npas4* may be followed by a second wave of expression of further effector genes related to glutamatergic pathways, GABAergic transmission and synaptic transmission, thereby accounting for the audiogenic seizure susceptibility in the GASH/Sal model.

### Deregulated Genes Involved in Calcium Channels

**FIGURE 8:**
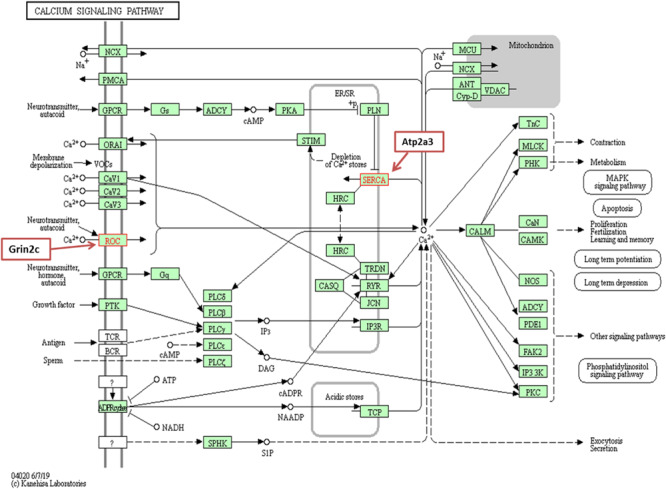
Calcium signaling pathway. *Grin2c* encodes an NMDAR channel that is associated with a ROC channel, and *Atp2a3* encodes a SERCA channel.

We observed underexpression of *Atp2a3* and *Grin2c* genes related to the calcium-signaling pathway in our comparative study of the IC transcriptomes in GASH/Sal *vs*. control hamsters after loud sound stimulation. The gene *Atp2a3* encodes a Ca^2+^ ATPase pump (SERCA), which actively re-accumulates released Ca^2+^ back into the sarco/endoplasmic reticulum, participating in the maintenance of Ca^2+^ homeostasis (Feng et al., 2013; Contreras-Leal et al., 2016). Moreover, this gene has been related to carcinogenic processes (Korošec et al., 2009), diabetes (Liang et al., 2011; Estrada et al., 2012) and epileptogenic processes (Kuzniewska et al., 2016; Lösing et al., 2017). On the other hand, the *Grin2c* encodes a subunit of the N-methyl-D-aspartate (NMDA) receptor. The receptor is a tetramer of different subunits (typically heterodimer of subunit 1 with one or more of subunits 2A-D, also named GRIN2A-D), forming a channel that have been related to receptor operated calcium channels (Ishi et al., 1993) and whose properties are determined by subunit composition (Figure 8).

Although *Grin2c* gene has not been related to epileptogenic processes, the subunit GRIN2A appears to be associated with the broadest and best characterized phenotypic spectrum, including a variety of disorders of the epilepsy aphasia spectrum and developmental and epileptic encephalopathy (Strehlow et al., 2019). In relation to these channels, it is known that the elevated intracellular calcium ion concentration activates processes such as hormone secretion, neurotransmitter release and calcium-dependent transcription of several genes, in addition to promoting spontaneous pacemaker activity in some neurons, muscles and secretory cells (Dolphin, 2016). Thus, the low expression of *Grin2c* observed in GASH/Sal animals after sound-induced seizures may contribute to Ca^2+^ deregulation of neuronal excitability and to the imbalance in intracellular Ca^2+^ as a result of changes in the formation of a receptor operated channel. Finally, with respect to the function of *Atp2a3*, calcium transport from the cytosol into the sarco/endoplasmic reticulum is decreased in the GASH/Sal after sound-induced seizures due to changes in the formation of SERCA channels. Most likely, both *Grin2c* and *Atp2a3* expression is downregulated in response to mutations of genes related to calcium response in the IC of the GASH/Sal.

### Deregulated Genes Involved in Potassium Channels

*Kcns1* and *Kcnj13* were overexpressed in the transcriptome of GASH/Sal animals when compared with the respective controls. *Kcns1* and *Kcnj13* genes encode voltage-dependent potassium channels and have been related to nociceptive signaling and retinal disturbance (Hao et al., 2013; Coussa et al., 2017). In particular, *Kcns1* encodes a potassium channel alpha subunit (Kv9.1) of the subfamily of electrically silent Kv (KvS) channel subunits (Kv5, Kv6, Kv8 and Kv9), which nevertheless generate functional channels at the plasma membrane when they heterotetramerize with Kv2 subunits. Interestingly, Kv9.1 was detected in the anterior and posterior ventral cochlear nucleus, and in the dorsal cochlear nucleus (Bocksteins, 2016). In addition, it has been suggested that the hyperpolarizing shift in voltage-dependent of Kv2.1 activation observed in rat neocortical pyramidal neurons could be caused by heterotetramerization with the KvS subunit Kv9.1 (Bocksteins, 2016). Moreover, Kv2.1 is associated with epilepsy-related mutations, thus raising the possibility that the epileptogenic effects on Kv2.1 may arise from an electrophysiological defect (Thiffault et al., 2015). In this context, *Knsc1* overexpression may be a mechanism of compensation for Kv2.1 channel over activation, thus explaining why the transcription of *Kcnb1*, which encodes Kv2.1, is not altered in GASH/Sal animals. Instead, Kcns1 expression is likely a target of gene expression modulation in response to *Kcnb1* mutations that cause epilepsy.

On the other hand, *Kcnj13* encodes channel Kir7.1, which is one of the most recently described members of the Kir channel super family expressed in brain, nephron, small intestine, and stomach (Pattnaik et al., 2012). A recent evidence indicates that mutant Kir7.1 channels are associated with inherited eye pathologies such as vitreo retinal degeneration and leber congenital amaurosis. Based on such finding, mutations implicated in channelopathies may result from the loss of function Kir7.1 channels (Coussa et al., 2017). Although Kir7.1 channels have not been directly associated with epilepsy, Winden et al. (2011) showed that *Kcnj13* was overregulated in chronic epilepsy. Consistent with this, our comparative transcriptome and RT-qPCR analyses of the IC in GASH/Sal and control hamsters suggest that *Kcnj13* overexpression following an epileptogenic event is a mechanism of compensation for Kir channel deregulation associated with epileptogenicity.

### Metabolomics Analysis

Our study showed that the gene expression of the nucleoside transporters SLC28 was overregulated in the GASH/Sal model, SLC28 and SLC29 are integral membrane proteins involved in the transport of nucleobases and nucleosides for the synthesis of nucleic acids (Pérez-Torras et al., 2013). The two families of nucleoside transporters SLC28 and SLC29 have several members that participates in modulating neurotransmission, vascular tone, immune responses and other physiological processes (Mulinta et al., 2017). The *Slc28a1* gene stands out as the protein-coding gene for Solute Carrier Family 28 Member 1 (Pastor-Anglada et al., 2005), and has been related to some pathologies such as cancer (Wang and Buolamwini, 2019), atrial fibrillation (Lin et al., 2016), and antiretroviral therapy absorption (Moketla et al., 2018), but not to epilepsy. Thus, *Slc28a1* overexpression in the IC of GASH/Sal animals in comparison with their controls may result from a process of physiological compensation whereby *Slc28a1* overexpression increases nucleic acid synthesis toward activating molecular processes to attenuate cellular stress, and consequently, contributing to epileptogenesis in the GASH/Sal.

On the other hand, it is known that epileptogenesis is associated with an increased, strong and persistent inflammatory state in the microenvironment of neural tissues, as is the case in subtle neuronal damage, gliosis and microgliosis (Alyu and Dikmen, 2017). Although the cellular and molecular mechanisms of epileptogenesis remain unclear, non-regulated focal or systemic inflammatory processes may lead to aberrant neuronal connectivity and hyperexcitable neural networks, which mediate the onset of epilepsy (Musto et al., 2016). In relation with this, our metabolomics analysis showed overrepresentation of inflammatory processes related to IL-4 and IL-13. These interleukins are well-known anti-inflammatory cytokines involved in cell repair and regeneration in inflammatory conditions (Kasaian et al., 2014). Therefore, the overrepresentation of this pathway may be associated with the aberrant onset of neuronal interactions underlying epilepsy, and the genes encoding of both interleukins can have the potential to be used as molecular biomarkers and targets for therapeutic approaches to epilepsy.

*Rxfp2* is another key gene that showed disrupted expression in our metabolomics analysis. In this case, *Rxfp2* was found underexpressed in GASH/Sal animals after sound-induced seizure when compared to their controls. The *Rxfp2* gene encodes a member of the G-protein-coupled receptor family and is expressed in sexual organs, kidney and brain; unsurprisingly, this gene has been related to alterations in the female and male reproductive system (Bathgate et al., 2006). According to Tomiyama et al. (2003), *Rxfp2* receptor downregulation and deactivation causes cryptorchidism, decreased spermatogenesis and male sterility. Our findings of *Rxfp2* downregulation are consistent with the results reported by Tomiyama et al. (2003), because GASH/Sal animals exhibit breeding difficulties, which may be influencing lineage of the species (unpublished data). However, to date, no direct relationship has been found between the *Rxfp2* gene and seizures.

### Genes Related to Epilepsy

The *C6* gene was overexpressed in the transcriptome analysis of GASH/Sal animals after audiogenic seizure. The C6 complement is one of the five plasma proteins incorporated into the potentially lytic terminal complement complex (Discipio and Gagnon, 1982; Walport, 2001). Since C6 is part of the membrane attack complex, this protein together with other complement components is involved in the destruction of susceptible cells and transmission of apoptosis signals (Muller-Eberhard, 1986). Furthermore, C6 deficiency has been implicated in decreased hemolytic activity (Dragon-Durey et al., 2003) and susceptibility to collagen antibody-induced lesions in arthritis (Banda et al., 2012). Interestingly, a study conducted by Buckingham et al. (2014) showed that deletion of the complement protein C5 significantly reduces the number of seizures in mice with the experimental cerebral malaria. Although no direct relationship has been found between C6 and epilepsy, our results indicated that it is worthwhile to explore whether the C6 contributes to seizure development as occurred with the C5 component. Based on the above, *C6* downregulation in the IC of GASH/Sal hamsters is likely related to membrane attack complex inhibition, which may act as a modulator after a seizure in this animal model.

Conversely, *Gadd45g* was found overexpressed in GASH/Sal with audiogenic seizures compared to control hamsters. *Gadd45g* is a member of a group of genes whose transcript levels increase in response to environmental stressors such as radiation or chemicals and which have been linked to cell cycle arrest, senescence, apoptosis, repair and demethylation of DNA, as well as functional maturation in various cellular systems, including the hematopoietic system. Although this gene in particular has not been implicated in epilepsy, studies focused on the serum response factor have shown *Gadd45g* overexpression after seizures in mouse pilocarpine epilepsy models (Lösing et al., 2017) and in human neocortical epilepsy (Beaumont et al., 2012). Therefore, *Gadd45g* overexpression may result from increased susceptibility to environmental stressors related to deregulated methylation processes passed along the GASH/Sal lineage.

Finally, *Ttr* was also overexpressed in GASH/Sal animals after audiogenic seizures. Plasma transthyretin (*Ttr*, previously termed prealbumin) is a 55k Da protein that participates in the transport of thyroxine and retinol plasma (vitamin A) (Herbert et al., 1986), and is usually located in the liver, central nervous system, and retinal pigmented epithelium (Soprano et al., 1985). In clinical studies, *Ttr* has been associated with familial amyloid polyneuropathy, an autosomal dominant multisystem neurological disease (Planté-Bordeneuve and Said, 2011), resulting from mutations that can cause seizures (Suhr et al., 2009; Franco et al., 2016). Thus, the overregulation of *Ttr* in the GASH/Sal model might affect the central nervous system, by producing seizures after loud sound stimulation. Nevertheless, *Ttr* gene was found overexpressed in the GASH/Sal also under free-seizure. Since a recent study reported that the GASH/Sal model carries two mutations in the *Ttr* gene that affect its structure and function (Díaz-Casado et al., 2020), our study indicated that the *Ttr* overexpression in the GASH/Sal might be compensatory mechanism to solve lack of functional TTR protein.

### Molecular Impacts of the Genes Deregulation in the IC

Audiogenic seizure rodent strains have been investigated worldwide to elucidate the neuronal and molecular mechanisms underlying seizure generation and propagation (Simler et al., 1999; Yechikhov et al., 2001; Garcia-Cairasco, 2002). Many similarities exist in the audiogenic seizure networks of various rodent models of audiogenic seizure, particularly regarding the critical role of the IC in seizure initiation, but how the audiogenic seizure susceptibility occurs and seizure arises is unknown (Faingold, 2004). Electrical stimulation of the lateral lemniscal pathways projecting to the IC evokes excitatory and inhibitory postsynaptic responses converging on a common neural population (Wagner, 1996; Moore et al., 1998; Ma et al., 2002). Most IC neurons receive both excitatory and inhibitory input from ascending fibers.

Glutamate is the primary excitatory transmitter throughout the IC (Adams and Wenthold, 1979; Bergman et al., 1989; Saint Marie, 1996). In addition, high levels of GABA exist in the IC (Tachibana and Kuriyama, 1974; Banay-Schwartz et al., 1989; Faingold et al., 1989) and many GABAergic neurons are likely to affect neuronal responses through intrinsic or commissural projections (Mugnaini and Oertel, 1985; Oliver et al., 1994). Based on this, our findings show deregulated gene expressions in the IC that might affect directly or indirectly the normal balance of excitatory and inhibitory conductance, resulting in neuronal network hyperexcitability and desynchronization.

In our experiments, the mRNA expression levels of *Egr3* was increased in the IC of the GASH/Sal, a result that was consistent with those obtained in previous studies (López-López et al., 2017). *Egr3* gene encodes a transcriptional factor that induce changes in *GABA_A_R* expression. This last gen encode a subunit of the GABA_A_ receptor (GABA_A_R), an ion channel that mediates the majority of inhibition in the central nervous system, and it has been associated with changes in GABA_A_R expression after the status epilepticus (Grabenstatter et al., 2012). Seizure induced transcriptional upregulation of the α4 subunit gene of the GABA_A_R (Roberts et al., 2005), which diminishes the effectiveness of GABA-mediated inhibition, and particularly, has been involved in the etiology of temporal lobe epilepsy (Grabenstatter et al., 2012). Moreover, within auditory pathways, the intrinsic electrical properties of neurons, and in particular their complement of potassium channels, play a key role in shaping the timing and pattern of action potentials produced by sound stimuli (Wu, 2005). The *Kcns1* gene (also known as Kv9.1) encodes a potassium channel alpha subunit that is expressed in a variety of neurons, including those of the IC, and is overexpressed in the GASH/Sal after the seizures. KCNS1 alters the kinetics and the voltage-dependence of activation and inactivation of KV2.1, a channel subunit that generates slowly inactivating delayed rectifier potassium currents. Because KV2.1 is expressed in IC (Hwang et al., 1992), this channel may be modulated by KCNS1, which might affect firing patterns significantly (Richardson and Kaczmarek, 2000).

In model neurons with rapidly inactivating inward current, the change in the voltage-dependence of activation produced by KCNS1 may allow the cells to follow high frequency stimulation more effectively (Richardson and Kaczmarek, 2000), which in turn might contribute to hyperexcitability through gene overexpression.

The disruption in gene expression of calcium and potassium channels, together with epileptogenesis-related genes that were found in our study, led us to infer that the altered gene expressions reported in the IC of the GASH/Sal might cause defects in biological process that contribute to its epileptogenic brain alterations. Future experiments that block the expression of these candidate genes and their correlations with changes in seizure severity will shed light on their role in epileptogenic mechanisms in the IC.

## Conclusion

Our data show gene deregulation in the IC of GASH/Sal animals compared to control hamsters. This genetic dysregulation involves 24 confirmed differentially expressed (know) genes and 7 unknown genes with undescribed sequences. Our findings suggest that audiogenic seizures are triggered in GASH/Sal hamsters through multiple molecular substrates, which activate several biological processes and metabolic pathways associated with epileptogenic events similar to those produced by tonic clonic seizures in humans. Therefore, we conclude that the GASH/Sal model could help to identify and characterize genes and pathways that were associated with seizures, whose could represent plausible antiepileptic drug targets.

## Data Availability Statement

Publicly available datasets were analyzed in this study. This data can be found here: https://www.ncbi.nlm.nih.gov/bioproject/230618.

## Ethics Statement

The animal study was reviewed and approved by Bioethics Committee of the University of Salamanca (approval number 300).

## Author Contributions

SD-R: validation, formal analysis, investigation, writing – original draft, visualization, conceptualization, software and data curation. DL-L, MH-T, and RG-N: writing – review and editing. AC-A: metabolomic analysis. DL: conceptualization, writing – review and editing, supervision, project administration and funding acquisition. All authors contributed to the revision of the manuscript and agreed with review the article.

## Conflict of Interest

The authors declare that the research was conducted in the absence of any commercial or financial relationships that could be construed as a potential conflict of interest.

## Footnotes

1 www.pantherdb.org

2 https://string-db.org

3 https://www.genome.jp/kegg

4 www.geneontology.org

5 https://reactome.org/

6 https://genecards.org/

## Supplementary Material

FIGURE S1 Detail of the NCBI GenBank database [https://submit.ncbi.nlm.nih. gov/subs/] showing the corresponding “project,” “sample,” and “experiment” inputs.

## Abbreviations

Actb: beta-actin
AP-1: Activator protein 1 Genes
*Atf3*: Activating transcription factor 3
*Atp2a3*: ATPase Sarcoplasmic/endoplasmic reticulum Ca^2+^ transporting 3
BDNF: Brain-derived neurotrophic factor
bp: Base pairs
C6: Complement C6
*Cd163*: CD163 molecule
cDNA: Complementary deoxyribonucleic acid
Ct: Mean threshold cycle
DNA: Deoxyribonucleic acid
*Egr1,2,3,4*: Early growth response 1, 2, 3 and 4 genes
FC: Fold change
*Fos, Fosb*: Fos-Fosb proto-oncogene
*Gadd45g*: Growth arrest and DNA damage inducible gamma
GASH/Sal: Genetic audiogenic seizure hamster from Salamanca
GENECARDS: The human gene database
*Gm12695*: Chromosome unknown C1orf87 homolog
GO: Gene ontological
*Grin2c*: Glutamate ionotropic receptor NMDA type subunit 2C
IC: inferior colliculus
IL-4, IL-13: Interleukin-4 and -13
*Junb*: Junb proto-oncogene
*Kcnj13*: Potassium voltage-gated channel subfamily J member 13
*Kcns1*: Potassium voltage-gated channel modifier subfamily S member 1
KEGG: Kyoto encyclopedia of genes and genomes
*Kir*: Inwardly rectifying K^+^ channels
*Kv*: Voltage-gated K^+^
logFC: Logarithm of Fold change
*Mmp3*: Matrix metallopeptidase 3
NMDAR: N-methyl -D-aspartate receptor
*Npas4*: Neuronal PAS domain 4
*Ogn*: Osteoglycin
PANTHER: Protein analysis through evolutionary relationships
Phred: Control of quality
*Rab29*: RAB29 Member RAS oncogene family
*Renbp*: Renin binding protein
ROC: receptor-operator channel
RNA: Ribonucleic acid
RT-qPCR: Quantitative reverse transcription real-time PCR
*Rxfp2*: Relaxin family peptide receptor 2
SERCA: Sarco/endoplasmic reticulum Ca^2+^ ATPase
SI: severity index
*Slc13a4*: Solute carrier family 13 member 4
*Slc28a1*: Solute carrier family 28 member 1
*Slc6a4*: Solute carrier family 6 member 4
*Sucnr1*: Succinate receptor 1
*Ttr*: Transthyretin
*Wdr38*: WD repeat domain 38.
