# Supplementary figures and images for: Inferior Colliculus Transcriptome After Status Epilepticus in the Genetically Audiogenic Seizure-Prone Hamster GASH/Sal
Source: Front Neurosci. 2020 May 26;14:508. doi: 10.3389/fnins.2020.00508 (PMC7264424; doi:10.3389/fnins.2020.00508)

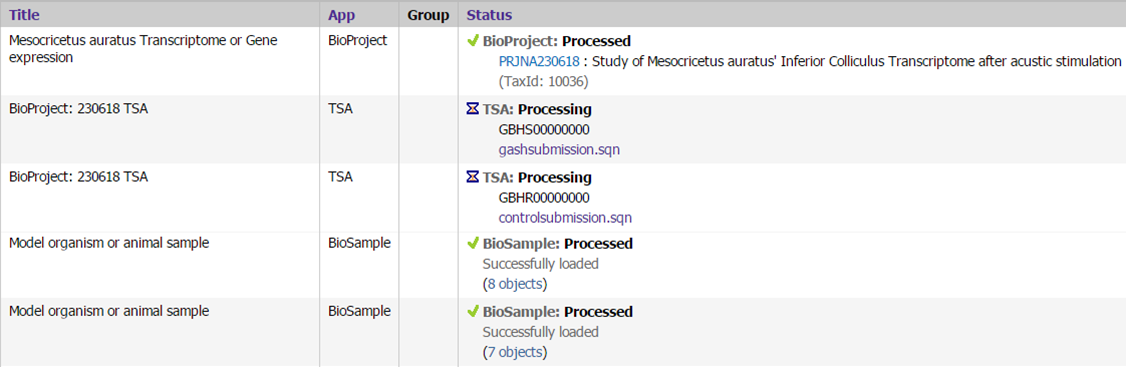

Supplement: FIGURE S1 — Detail of the NCBI GenBank database [https://submit.ncbi.nlm.nih. gov/subs/] showing the corresponding “project,” “sample,” and “experiment” inputs. [file Image_1.TIF]
